# Measuring associations between the microbiota and repeated measures of continuous clinical variables using a lasso-penalized generalized linear mixed model

**DOI:** 10.1186/s13040-018-0173-9

**Published:** 2018-06-15

**Authors:** Laura Tipton, Karen T. Cuenco, Laurence Huang, Ruth M. Greenblatt, Eric Kleerup, Frank Sciurba, Steven R. Duncan, Michael P. Donahoe, Alison Morris, Elodie Ghedin

**Affiliations:** 1 Department of Computational & Systems Biology, University of Pittsburgh School of Medicine, Pittsburgh, PA 15261 USA; 2 Department of Biology, Center for Genomics & Systems Biology, New York University, New York, NY 10003 USA; 3 Genentech, 1 DNA Way, MS-231C, South San Francisco, CA 94080 USA; 4 Department of Human Genetics, Graduate School of Public Health, University of Pittsburgh, Pittsburgh, PA 15261 USA; 5 Department of Medicine, School of Medicine, University of California, San Francisco, CA 94143 USA; 6 Departments of Clinical Pharmacy, Epidemiology and Biostatistics, Schools of Pharmacy and Medicine, University of California, San Francisco, CA 94143 USA; 7 Department of Medicine, David Geffen School of Medicine, University of California, Los Angeles, CA 90095 USA; 8 Division of Pulmonary, Allergy, and Critical Care Medicine, Department of Medicine, University of Pittsburgh School of Medicine, Pittsburgh, PA 15213 USA; 9 College of Global Public Health, New York University, New York, NY 10003 USA

**Keywords:** Microbiota, 16S, ITS, Repeated measures, Continuous outcomes, GLMM, Lasso

## Abstract

**Background:**

Human microbiome studies in clinical settings generally focus on distinguishing the microbiota in health from that in disease at a specific point in time. However, microbiome samples may be associated with disease severity or continuous clinical health indicators that are often assessed at multiple time points. While the temporal data from clinical and microbiome samples may be informative, analysis of this type of data can be problematic for standard statistical methods.

**Results:**

To identify associations between microbiota and continuous clinical variables measured repeatedly in two studies of the respiratory tract, we adapted a statistical method, the lasso-penalized generalized linear mixed model (LassoGLMM). LassoGLMM can screen for associated clinical variables, incorporate repeated measures of individuals, and address the large number of species found in the microbiome. As is common in microbiome studies, when the number of variables is an order of magnitude larger than the number of samples LassoGLMM can be imperfect in its variable selection. We overcome this limitation by adding a pre-screening step to reduce the number of variables evaluated in the model. We assessed the use of this adapted two-stage LassoGLMM for its ability to determine which microbes are associated with continuous repeated clinical measures.

We found associations (retaining a non-zero coefficient in the LassoGLMM) between 10 laboratory measurements and 43 bacterial genera in the oral microbiota, and between 2 cytokines and 3 bacterial genera in the lung. We compared our associations with those identified by the Wilcoxon test after dichotomizing our outcomes and identified a non-significant trend towards differential abundance between high and low outcomes. Our two-step LassoGLMM explained more of the variance seen in the outcome of interest than other variants of the LassoGLMM method.

**Conclusions:**

We demonstrated a method that can account for the large number of genera detected in microbiome studies and repeated measures of clinical or longitudinal studies, allowing for the detection of strong associations between microbes and clinical measures. By incorporating the design strengths of repeated measurements and a prescreening step to aid variable selection, our two-step LassoGLMM will be a useful analytic method for investigating relationships between microbes and repeatedly measured continuous outcomes.

**Electronic supplementary material:**

The online version of this article (10.1186/s13040-018-0173-9) contains supplementary material, which is available to authorized users.

## Background

Epidemiologic studies, ranging from clinical trials to observational studies, often include the collection of demographic, disease symptoms, treatment, diagnostic tests and clinical laboratory information. Recent evidence that the human microbiome influences disease occurrence [1, 2] has led to interest in how the microbiome may more generally impact clinical and treatment outcomes, and the natural history of a disease. While continuous clinical measures are used to describe and to identify risk subgroups in the patient population, the relationship between these measures and the microbiome is less often examined. This rarity is in part caused by methodology limitations in applying current microbiome and analytic techniques to continuous clinical data.

One stumbling block to analyzing the microbiome in the context of clinical variables comes from repeated measurements, i.e. the same measurement taken at multiple time points or multiple measurements made at a single time point. Even in non-equilibrated communities, where variance between repeated measures is high, measurements of the microbial community are expected to be highly correlated with each other, thus presenting a problem for standard statistical methods. However, repeated measures can provide important data for processes that evolve or change over time. Techniques to analyze repeated measures would be of use to the microbiome field as repeated measurements are often necessary to obtain a more complete understanding of a system of interest.

An additional challenge in analyzing clinical outcomes and biomarkers in the context of the microbiome is that the outcomes are often continuous rather than dichotomous variables. Continuous variables are those that can take on any value within a given range; when they are converted to a categorical or dichotomous format, in some instances, information is lost. In practice, count variables, although not technically continuous, are treated as continuous variables. These continuous variables, as opposed to categorical variables, have repeatedly been dichotomized in the microbiome literature [3, 4] with the potential for loss of nuance in the relationship between the variable and the microbiota.

Our primary interest was in assessing genus or species abundance as a potential predictor of clinical laboratory and other measures that have repeated measurements. We focused on the generalized linear mixed model (GLMM) method because it handles both continuous outcome variables and repeated measures. GLMMs have just recently been incorporated into microbiome studies [1, 5, 6]. These early adopters of the GLMM methods primarily use binary or categorical groups (e.g. sample site, treatment, pregnant/non-pregnant) to explain the outcome of interest, such as species abundance. When combined with a penalty parameter—an additional term that eliminates extraneous explanatory variables—species abundance can instead be used to explain clinical outcomes, including continuous clinical measurements.

Mixed models—both GLMMs and linear mixed models—have been used in ecology without penalty parameters at least as long as next generation sequencing-based microbiome studies have existed [7]. Mixed models incorporate both fixed effects that are the same for every observation or sample, and random effects that apply to select samples or groups of samples. Through the use of random effects, linear mixed models are designed to handle repeated measures and other complex study designs [7]. In addition, GLMMs attempt to model data that do not follow a traditional normal distribution. The linear relationship between the outcome and predictors is redefined as the set of linear predictors and their relationship to the expected value of the outcome via a “link” function. This link function, along with the variance of the expected value of the outcome, is selected from the members of the exponential distribution family.

A complementary analytic method, penalized regression, has been used in genomics and metagenomics for several years [8]. One of the common penalties used in these studies is the lasso (or L1) penalty, which has the advantage of performing variable selection by reducing some coefficients to zero. The variables whose coefficients are reduced to zero can be removed from the model without impacting the model’s ability to predict the outcome of interest. The elastic net penalty, which is the combination of the lasso penalty and the alternative ridge (or L2) penalty, reduces some coefficients to zero and shrinks others, but not to zero, limiting its capacity to perform variable selection [9]. Only lasso performs variable selection without having to select a coefficient size threshold to define association, but it has limitations when handling many variables that are correlated with each other.

The lasso penalized generalized linear mixed model (LassoGLMM), developed in 2011 for sports statistics and human-computer interactions [10, 11], has properties that make it well-suited for microbiome applications. This model leverages the power gained by repeated measures and compensates for the large number of variables by combining the lasso penalty with GLMMs. The lasso penalty resolves the problem of having many more explanatory variables than observations by forcing some coefficients to be equal to zero and leaving only those variables (or in our case, microbes) with the strongest associations with non-zero coefficients. Repeated measures can be incorporated through LassoGLMM mixed effects via a random effect for each patient and repeated measurement.

We present a two-stage approach that couples a correlation-based screening step with the LassoGLMM to examine the relationships between the microbiota and continuous variables related to health and inflammation. The data originate from two clinical studies of the respiratory tract: a 16S rRNA gene survey of the oral microbiota from the Oral Cyclosporine in Chronic Obstructive Pulmonary Disease study (OC-COPD; clinicaltrials.gov ID: NCT00974142, a randomized controlled clinical trial), and a combination bacterial 16S rRNA gene and fungal Internal Transcribed Spacer (ITS) analysis of the bronchoalveolar lavage (BAL) for the Pittsburgh site of the Lung HIV Microbiome Project (LHMP; clinical trials ID: NCT00870857, an observational cohort study). In the OC-COPD study, we sought to discover associations between the oral microbiota and laboratory values measured in peripheral blood. In the LHMP, we aimed to identify which bacteria and fungi were associated with increased inflammation both locally in the lungs and systemically in the blood.

## Methods

Multiple specimens including oral washes and BAL for microbiota characterization, and blood for chemistry, inflammatory markers, and other laboratory measurements were collected as part of the OC-COPD and the LHMP. The OC-COPD dataset included 15 samples from eight individuals at pre-randomization (trial week 0) and at trial week 16 (one participant did not have a sample for the pre-randomization visit). These OC-COPD participants, who were sequentially enrolled from the parent trial, had advanced COPD but were free of active infections. Specific inclusion criteria included: 45–80 years of age, presence of advanced COPD (defined as forced expiratory volume in 1 s, FEV1, between 25 and 60% predicted), and non-responsive to traditional inhaler therapy. Once enrolled, participants were randomized to receive for 16 weeks the test drug, cyclosporine (an immune suppressant), or a placebo (additional eligibility requirements for the trial are described at clinicaltrials.gov, identifier NCT00974142). Laboratory outcomes include 32 blood measurements found in a typical blood chemistry panel with electrolytes. Clinical independent variables used were gender and treatment group (test drug or placebo).

The LHMP lung microbiome dataset contained 30 samples from 21 participants who had BAL performed on their right and left lungs at the same clinical visit. This group included both HIV-infected (HIV+; *N* = 11) and HIV-uninfected (HIV-; *N* = 10) individuals, classified as current smoker (*N* = 3), former smokers (defined as having quit more than 6 months prior to the study; N = 3), and never smokers (defined as having smoked fewer than 100 cigarettes in a lifetime; *N* = 15). Inclusion criteria included no use of antibiotics in the past 3 months and no evidence of acute respiratory disease for 4 weeks. The lung microbiome was sampled by BAL following an oral wash and gargle with antiseptic mouthwash. Specific inclusion criteria and sampling procedures can be found in [12]. The 16S rRNA gene and ITS rRNA sequence data are described in [12, 13], respectively. Laboratory outcome variables include 12 cytokines measured in both the BAL and the blood. Six cytokines that were not detectable in 90% of the samples were excluded from further analysis. Clinical independent variables used were HIV status and smoking history category.

### Sequence data processing

The sample processing procedures were performed as previously described in [12, 13]. In brief, all samples had DNA extracted using standard techniques with the PowerSoil® DNA Isolation Kit from MO BIO (Carlsbad, CA). For the OC-COPD, the bacterial V4 hyper-variable region of the 16S rRNA gene was amplified and sequenced on the Illumina MiSeq platform. For the LHMP, the hyper-variable regions 1 through 3 (V1-V3) were amplified and sequenced using the Roche 454 GS-FLX platform with Titanium chemistry. For fungal DNA sequencing, the ITS1 was amplified and sequenced on the Ion PGM™ Sequencer using the 400 bp protocol [14]. Sequences were processed using the QIIME pipeline version 1.7 [15] with default settings for de novo Operational Taxonomic Unit (OTU) picking. Bacterial 16S rRNA gene sequences were clustered at 97% similarity and fungal ITS sequences were clustered at 99% similarity. Additional processing and taxonomic assignment for the ITS sequences was performed using FHiTINGS [16]. Samples with fewer than 1000 16S rRNA gene (bacterial) reads, and samples with fewer than 100 ITS (fungal) reads were considered to have failed and were removed from further analysis. All OC-COPD samples were considered successfully sequenced but 16 LHMP samples were considered to have failed ITS sequencing.

After initial taxonomic assignments were made using the default settings in QIIME or FHiTINGS, OTUs were combined by taxonomic assignment at the genus level. For each kingdom, all genera counts were normalized using total sum scaling, also known as relative abundance. Any bacterial genus present in less than half of the samples or any fungal genus present in fewer than 10% of samples was removed.

### Analytic association stage 1: Variable screening step

**Fig. 1:**
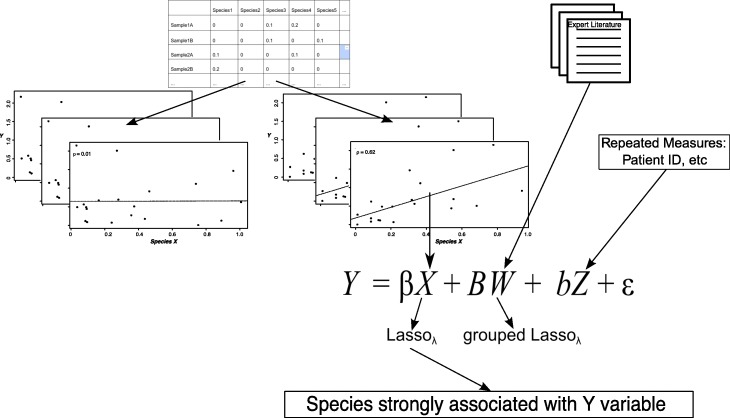
Overview of the two-step LassoGLMM model developed. Species (or genera, OTUs, or any other explanatory variables of interest) are divided into those that are correlated with the dependent continuous variable, *Y*, and those that are not. Species that are correlated are stored in a matrix *X*. Relevant categorical variables, found through a review of expert literature or other means, are stored in a matrix *W*. Indicators of repeated measures such as patient ID are stored in matrix *Z*. Matrices *X, W,* and *Z* are entered into a generalized linear mixed model to be regressed on outcome variable *Y*. Coefficient β for matrix *X* and coefficient *B* for *W* are subjected to the lasso penalty. Any species that retain non-zero coefficients are considered strongly associated with the dependent variable *Y*

The number of genera present is often at least an order of magnitude larger than the number of subjects sampled and presents an analytic challenge due to sparse microbial data. We overcome this problem by preceding LassoGLMM regression with a variable screening step based on correlation. We calculated the Spearman correlation for each clinical response-microbial genera pair, and used the microbial genera with significant Spearman correlations (*p* < 0.05 without multiple testing correction) as independent variables in the regression model for that clinical response. Figure 1 shows an overview of this two-step method.

### Analytic association stage 2: Lasso-penalized generalized linear mixed model

The microbial genera that pass the analytic association stage 1 screening are input into the LassoGLMM as independent variables (*X*) in eq. 1 below. The LassoGLMM combines variable selection with the flexibility to account for repeated measures and other random effects. It can be built up from the random-intercept linear mixed model (eq. 1):

$$ Y=\beta X+ bZ+\varepsilon $$

where *Y* is the response variable, or outcome of interest, *X* is the matrix of the fixed effects including genera abundances, *Z* is the matrix of the random effects including patient, ε is the random error, and β and b are the coefficient vectors corresponding to fixed and random effects. For example, we modeled the response variable, *Y*, of blood glucose on the relative abundance of bacterial genera in the mouth, formatted as matrix *X*, while accounting for the individual participant as a random effect, *Z*. Here, genera abundances and disease status are treated as fixed. Each study participant or time point is treated as random effects. The fixed effects, *X*, can be redefined into continuous (*X*) and categorical (*W*) variables (eq. 2):

$$ Y=\beta X+ BW+ bZ+\varepsilon $$

*W* is the matrix form of the ‘dummy’ variables indicating each level among the categorical variables including disease status. This split is important for the penalization of the categorical variables and described below.

For OC-COPD and LHMP data analyses, random effects and categorical variables (fixed effects) were study specific. In the OC-COPD study, an individual was sampled at a pre-randomization visit and 16 weeks later. The visit time point was analyzed as a random effect to account for any seasonal or batch processing effects. In the LHMP study, the right and the left lungs were sampled in the same individual during the same visit in a randomized order (right first or left first). An indicator of this order was included as a random effect to account for any order bias, including the possibility of contamination from the upper respiratory tract in the first side. In our regression models we included the following categorical variables that are known to be associated with the outcomes (*Y*) of interest: gender [17] and treatment (drug or placebo) in the OC-COPD models; smoking [18] and HIV [19] status for the LHMP models.

By their nature, many of the variables (genera or OTUs) in the microbiota are highly correlated with each other. This correlation makes including all variables in the regression redundant and necessitates the use of the lasso or other penalty, which can select the variables most strongly associated with the outcome of interest. The penalty parameter λ performs variable selection by forcing the smaller β and *B* coefficients to equal zero during the maximal likelihood estimation of the coefficients in eq. 2. All of the *B* values of one categorical variable are penalized together with a grouped lasso penalty adapted from [20]. Thus, either all possible indicators for a categorical variable are included in the model, or none are included. For example, the LHMP smoking statuses ‘current’, ‘former’, and ‘never’ result in two dummy variables, one for ‘current’ and one for ‘former’. The *B* coefficients for both dummy variables are either reduced to zero or both included in the model. By increasing λ, more of the fixed effect coefficients will be forced to zero. It is important to note that only the fixed effects coefficients are subject to the lasso penalty. Random effects are included in the model regardless of the size of λ.

The optimal lasso penalty term (λ) was selected for each model by scanning between 0 and 200 (by increments of 1) using the R package *glmmLasso* version 1.3.3 [21], and identifying the model with the lowest Bayesian Information Criterion (BIC) [21] as optimal. When λ = 0, if the Fisher matrix was not invertible (i.e. the regression could not be completed) we started the scan at λ = 1. We considered those genera with non-zero coefficients in the model using the optimal penalty term to be associated with the response variable. Following Groll’s recommendation [10], we then ran a GLMM regression including only those genera with non-zero coefficients using the R package *lme4* [22]. This final regression step is related to the adaptive lasso penalty and is designed to compensate for the lack of oracle properties of the basic lasso penalty that we used here [11]. Oracle properties are the features of a regression model that ensure it reproducibly estimates the correct coefficients. The LassoGLMM lacks these properties but by running a final regression, the reproducibility and accuracy of the coefficients are improved. The results of this final regression led to the detection of an association, and if that association was positive (more microbes when the variable is high), or negative (more microbes when the variable is low). A list of all final models is presented in Table 1.

**Table 1:** Outcome variables and model abbreviations. A list of all final models by outcome variable, and a short abbreviation based on which study the model originated from

Outcome variable	Study	Abbreviation
Percent Neutrophils	OC-COPD	O1
Blood Urea Nitrogen (BUN)	OC-COPD	O2
Immunoglobulin-M (IGM)	OC-COPD	O3
Partial Pressure of Oxygen (PPO)	OC-COPD	O4
SAT	OC-COPD	O5
Alkaline Phosphatase	OC-COPD	O6
Serum Glutamic Oxaloacetic Transaminae (SGOT)	OC-COPD	O7
Serum Glutamic-Pyruvic Transaminase (SGPT)	OC-COPD	O8
Cholesterol	OC-COPD	O9
Glucose	OC-COPD	O10
Bronchoalveolar Lavage (BAL) Interleukin Receptor Antagonist (IL-ra)	LHMP	L1
Systemic Interleukin Receptor Antagonist (IL-ra)	LHMP	L2

### Evaluating models

We evaluated the fit for each of our mixed models using both the marginal and conditional R^2^ coefficients of variation [23]. Marginal R^2^ represents the percent of variation explained by the fixed effects while conditional R^2^ represents the variation explained by the entire mixed model, both fixed and random effects. Compared to the BIC that was used for penalty optimization, the coefficients of variation provide a more absolute measure of the goodness of fit for the model in question that can be compared across models. We also inspected the residual plots to ensure that the relationship between the microbes and clinical variables was linear. When a relationship was found to be non-linear, we attempted to refit the model with a generalized model to account for the potentially non-normal distribution of outcome variable.

### Comparison to a dichotomous method

Because there is no single best way to evaluate the association between microbiota abundance and a continuous variable, we compared our LassoGLMM method to dichotomizing the outcome of interest into two groups and comparing the genera abundance between groups. For the comparison we used a basic dichotomous variable method, the Wilcoxon (or Mann-Whitney U) test [24]. The Wilcoxon test is a non-parametric statistical test that compares rank statistics between two groups. To dichotomize our data, we divided samples into those above and those below the sample average for the outcome of interest.

## Results

### Associations between bacteria and laboratory measurements

To identify associations between the easily accessible oral bacteria and laboratory values measured in blood, we characterized the microbiota in 15 oral wash samples from eight individuals at two different time points, 16 weeks apart. A metabolic panel of 32 measurements, including electrolytes and cholesterol levels, was performed at each visit. In the 15 oral washes, we found a total of 95 bacterial genera present in at least half the samples. All samples contained *Streptococcus* (mean: 32.2%, standard deviation: 11.6), *Prevotella* (mean: 12.4%, SD: 6.5), *Rothia* (mean: 10.6%, SD: 6.5), *Fusobacterium* (mean: 6.2%, SD: 5.0), and *Veillonella* (mean: 5.6%, SD: 3.7).

We calculated Spearman correlations between every pair of bacterial genera and blood metabolic profile measurement. There were 202 correlations (out of 1425 possible, 14.2%) that were nominally significant, *p* < 0.05 before correcting for multiple hypotheses testing. Each clinical variable was significantly correlated with 1 to 20 genera, averaging 7.5 nominally significant correlations per clinical outcome (Additional file 1: Table S1). Out of the 95 genera, 75 were nominally significantly correlated with 1 to 9 of the clinical variables.

**Fig. 2:**
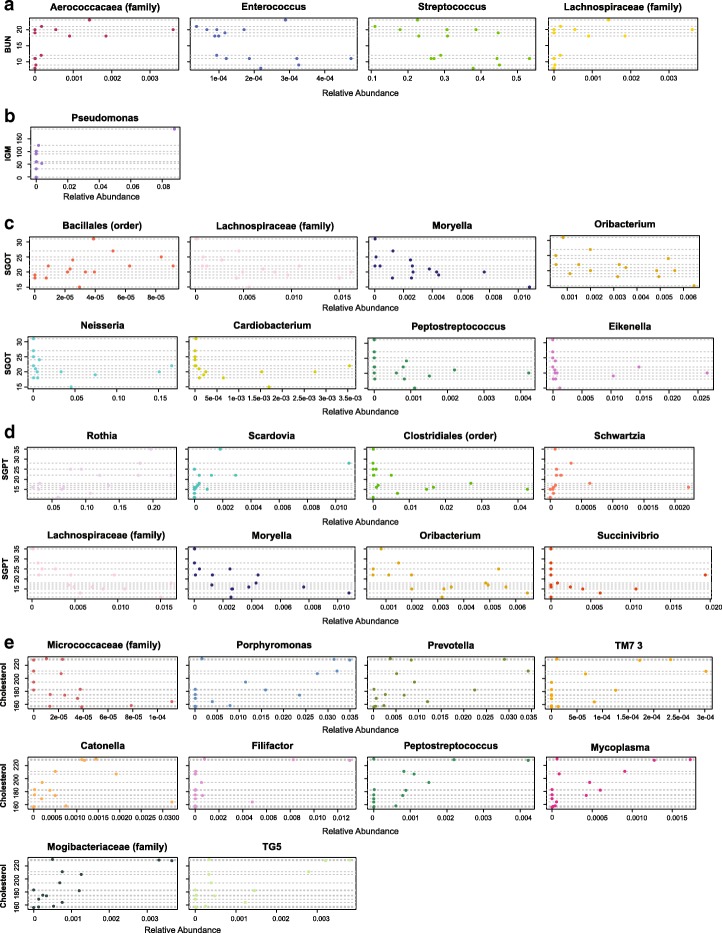
OC-COPD associations between laboratory measurements and bacteria identified by LassoGLMM. Strong associations between bacteria and (**a**) BUN (O2), (**b**) IGM (O3), (**c**) SGOT (O7), (**d**) SGPT (O8), and (**e**) cholesterol (O9). Each horizontal grey line represents an individual. When a colored circle is located on the grey line, it is the relative abundance of that microbe for that subject. Perfect positive association between clinical variable and bacteria would form a line from the bottom-left to the top-right of the graph and would have a highly positive β coefficient in the LassoGLMM. Perfect negative association would form a line from the top-left to the bottom-right of the graph and would have a highly negative β coefficient

The genera that had a nominally significant correlation with a clinical variable were entered as potential explanatory variables into a LassoGLMM to predict that clinical variable along with Cyclosporine/placebo treatment assignment and gender. All but 64 genera coefficients (out of the 202 nominally significant correlations) were forced to zero by the lasso penalty indicating no association with the clinical outcome measure. The 64 coefficients that were not forced to zero were considered strong associations (Table 2). Ten laboratory measures (Table 1) were associated with bacterial genera since their models retained non-zero coefficients (see Fig. 2, Additional file 2: Figure S1): percent neutrophils (model O1), blood urea nitrogen (BUN) (model O2), immunoglobulin M (IGM; model O3), partial pressure of oxygen (model O4), SAT (model O5), alkaline phosphatase (model O6), serum glutamic oxaloacetic transaminase (SGOT; model O7), serum glutamic-pyruvic transaminase (SGPT; model O8), cholesterol (model O9), and glucose (model O10). Of these lab measures, BUN, IGM, partial pressure of oxygen, SAT, and SGPT (models O2, O3, O4, O5, and O9) were strongly associated with all of the bacterial genera that correlated with the measurement (optimal penalty parameters of 0). For the remaining 5 models, the optimal λ penalty parameter ranged from 2 to 144. In each of these 5 models, higher λ penalty parameter values revealed no association (*β* coefficient reduced to 0) between the outcome of interest and some of the bacterial genera that were correlated with it, when the other correlated bacterial genera were accounted for in the model. The higher λ penalty parameter values also revealed no association (*B* coefficient reduced to 0) between glucose and drug treatment assignment in model O10 or between cholesterol and gender in model O9.

**Table 2:** Laboratory measurements and their strongly associated bacteria in OC-COPD. Bacteria that could not be classified to the genus level are listed at the lowest taxonomic level that could be confidently identified. Bacteria in bold are negatively associated with the laboratory measurement, indicating that higher microbial abundance is associated with lower measurement level

Percent Neutrophils (O1)	BUN (O2)	IGM (O3)	Partial Pressure of Oxygen (O4)	SAT (O5)	Alkaline Phosphatase (O6)	SGOT (O7)	SGPT (O8)	Cholesterol (O9)	Glucose (O10)
***Bacteroidales*** **(order)**	***Aerococcaceae*** **(family)**	*Pseudomonas*	***Bacillus***	***Bacillus***	***Bifidobacteriaceae*** **(family)**	***Bacillales*** **(order)**	***Rothia***	***Micrococcaceae*** **(family)**	*Rothia*
*S-24* (family in *Bacteroidales* order)	***Enterococcus***		*Pseudomonas*	*Pseudomonas*	***Weeksellaceae*** **(family)**	***Lachnospiraceae*** **(family)**	***Scardovia***	*Porphyromonas*	***Porphyromonas***
*Clostridiaceae*	***Streptococcus***				***Gemellales*** **(order)**	***Moryella***	*Clostridiales* (order)	*Prevotella*	***Tannerella***
*Oribacterium*	*Lachnospiraceae* (family)				***Gemellaceae*** **(family)**	*Oribacterium*	***Lachnospiraceae*** **(family)**	*Catonella*	***Prevotella***
*Oscillospira*					***Granulicatella***	*Peptostrepto-coccus*	***Moryella***	*Filifactor*	***Gemellaceae*** **(family)**
*Ruminococcus*					*Lactobacillus*	***Eikenella***	*Oribacterium*	*Peptostreptococcus*	*Lactobacillus*
*Phascolactobacterium*					*Eikenella*	***Neisseria***	***Schwartzia***	*Mogibacteriaceae* (family)	***Peptostrepto-coccaceae*** **(family)**
*Succinivibrio*					***Neisseria***	***Cardiobacterium***	*Succinivibrio*	*TG-5* (member of *Dethiosulfovibronaceae* family)	***Peptostrepto-coccus***
					*Aggregatibacter*			*TM-7.3*	*Veillonella*
								*Mycoplasma*	***Mogibacteriaceae*** **(family)**
									***TG-5*** **(member of** ***Dethiosulfovibro-naceae*** **family)**
									***Mycoplasma***

### Associations between bacteria or fungi and cytokines

Using the LHMP dataset, we sought to identify associations between indicators of local or systemic inflammation and bacteria and/or fungi detected in BAL samples. We used bacterial and fungal surveys previously performed on 30 BAL samples from 21 individuals [12, 13]. Across all samples we found 49 bacterial genera in at least half of the samples and 28 fungal genera in at least 10% of the samples. There were 106 Spearman correlations (out of 1386 possible, 7.6%) that were nominally significant at *p* < 0.05. Each cytokine had between 2 and 9 nominally significant correlations with bacterial and fungal genera (average number of genera nominally correlated with each cytokine = 5.9) (Additional file 3: Table S2). Conversely, of the 77 genera identified, 42 were nominally significantly correlated with 1 to 7 cytokines.

**Fig. 3:**
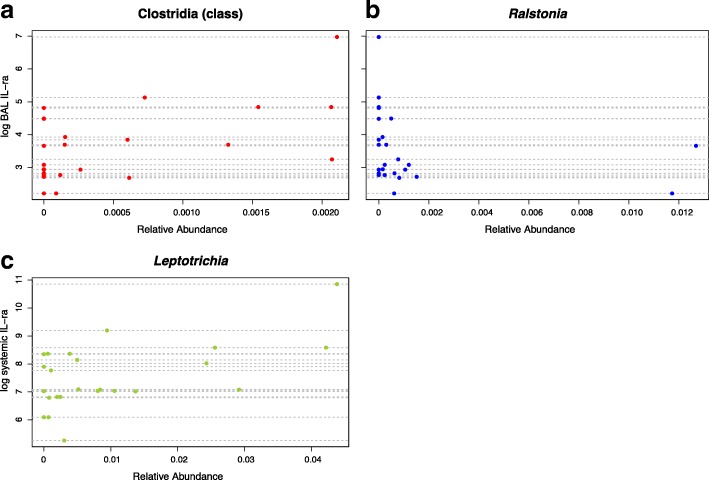
LHMP associations between cytokines and bacteria and fungi identified by LassoGLMM. Strong associations between bacteria and (**a**) BAL IL-ra (L1) and (**b**) systemic IL-ra (L2). Each horizontal grey line represents a subject. When a colored circle is located on the grey line, it is the relative abundance of that microbe for that individual. Perfect positive association between cytokine and bacteria or fungi would form a line from the bottom-left to the top-right of the graph and would have a highly positive β coefficient in the LassoGLMM. Perfect negative association would form a line from the top-left to the bottom-right of the graph and would have a highly negative β coefficient

These bacterial and fungal genera were entered into the LassoGLMM along with HIV status and smoking status as potential explanatory variables. As in the oral microbiota model evaluations, most genera coefficients (103 out of 106) in the LHMP models were reduced to zero by the lasso-penalty, indicating no associations between the genera and the outcome of interest. All fungal genera coefficients in all models were reduced to zero. The three bacterial genera that maintained non-zero coefficients are presented in Table 3. In models for the 16 other cytokines (see Additional file 3: Table S2 for a list of all cytokines analyzed), all genera coefficients were forced to zero while HIV and/or smoking status coefficients were non-zero, which indicates they may explain variation in the cytokine levels better than any components of the microbiota. Two models had evidence of strong genera association with non-zero coefficients (Fig. 3), BAL interleukin receptor antagonist (IL-ra) (model L1), and systemic IL-ra (model L2). BAL IL-ra (model L1) had an optimal penalty parameter of 0, indicating that all correlated bacteria (no fungi were nominally significantly correlated with BAL IL-ra) were strongly associated with BAL IL-ra. Conversely, systemic IL-ra (model L2) had an optimal penalty parameter of 13, retaining one bacterial genus as strongly associated and eliminating seven others as well as HIV and smoking status.

**Table 3:** Cytokines and their strongly associated microbes in LHMP. Bacteria and fungi that could not be classified to the genus level are listed at the lowest taxonomic level that could be identified. Microbe in bold is negatively associated with the cytokine, indicating that higher microbial abundance is associated with lower cytokine level

BAL IL-ra (L1)	Systemic IL-ra (L2)
*Clostridia* (class)	*Leptotrichia*
***Ralstonia***

### Model evaluation

**Fig. 4:**
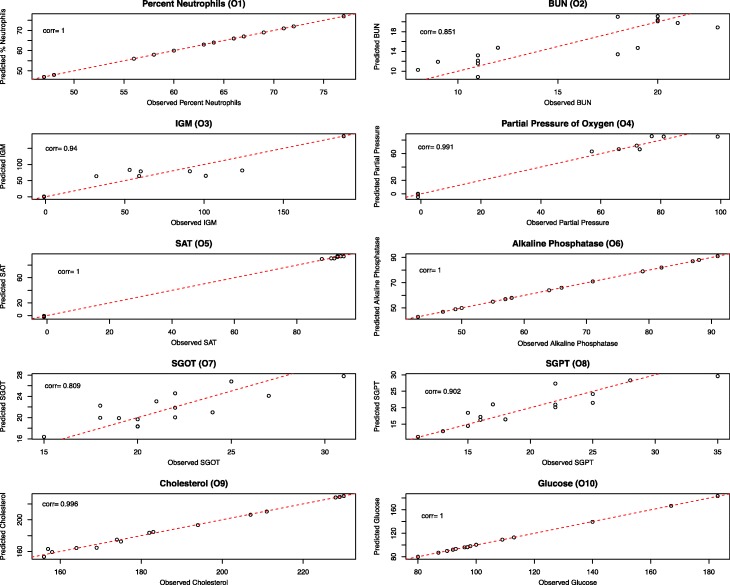
Observed vs predicted value plots evaluating the fit of the LassoGLMMs from the OC-COPD study. Each plot represents one LassoGLMM with non-zero coefficients. The value observed (*X*-axis) is plotted against the value predicted by the LassoGLMM (*Y*-axis). Each point represents a sample. The red line indicates where the predicted value matches the observed value. For models that deviate from this line (O2, O3, and O7), we attempted to fit a generalized model but found no significant improvements in fit

To evaluate our models, we used both marginal (fixed effects only) and conditional (both fixed and random effects) coefficients of determination, or R^2^ [23]. For GLMMs based on models O1-O10 we had an average marginal R^2^ value of 0.44 (SD 0.32) and an average conditional R^2^ value of 0.90 (SD 0.14; Table 4). These R^2^ values demonstrate that, on average, 44% of the variation seen in the clinical variables was explained by the bacteria that were strongly associated with the laboratory measurement, gender, and drug treatment, and that our whole model explained 90% of the variation seen in the clinical variables. However, models O1, O4, O5, O6, and O10 were found to be over-fitting the data with conditional R^2^ greater than 0.99. Both GLMMs based on the LHMP models, L1 and L2, were also found to be over-fitting the data with conditional R^2^ equal to 1.00. The residuals from the remaining models fit the data reasonably well (Fig. 4). The most notable exception was in model O3, for IGM, which has large residuals whose pattern indicates a non-linear relationship. We attempted to fit a generalized model to these data to allow for the non-linear relationship and potentially non-normally distributed outcome, as well as to models O2 and O7, but were unable to significantly improve the fit based on residual inspection (data not shown).

**Table 4:** Marginal and conditional coefficients of variation (R^2^) for OC-COPD models and Lasso-penalized GLMM variants. The two-step LassoGLMM method, in columns 1 and 2, is presented here. The original LassoGLMM, in columns 3 and 4, omits the first step of correlation-based variable screening, adding all OTUs to the LassoGLMM. The GLMM with correlated genera, in columns 5 and 6, uses the correlation-based variable screening step, adding only those variables that are correlated with the outcome to the model, but modifies the second step to not include the lasso penalty. Each method column contains the marginal and conditional R^2^ that represent fit of the fixed effects and entire model, respectively

	Two-step LassoGLMM		Original LassoGLMM		GLMM with correlated genera	
	Marginal R^2^	Conditional R^2^	Marginal R^2^	Conditional R^2^	Marginal R^2^	Conditional R^2^
BUN (O2)	0.58	0.60	No non zero coefficients		All correlated variables were in Two-step LassoGLMM	
IGM (O3)	0.19	0.89	No non zero coefficients		All correlated variables were in Two-step LassoGLMM	
SGOT (O7)	0.22	0.84	No non zero coefficients		0.50	0.59
SGPT (O8)	0.44	0.75	No non zero coefficients		All correlated variables were in Two-step LassoGLMM	
Cholesterol (O9)	0.80	0.93	0.95	0.98	0.99	1.00

We then compared our models from two-step LassoGLMMs with 1) LassoGLMMs, as originally described by Groll [10, 21], which does not include variable screening, and with 2) a two-step non-penalized GLMM that uses all correlated genera that passed variable screening as explanatory variables. This latter model is our two-step LassoGLMM with a λ penalty parameter of 0. The marginal and conditional R^2^ values for the three model styles are included in Table 4. With the notable exception of model O9, we found that our two-step model performed at least as well as the original LassoGLMM without a variable screening step and as the two-step non-penalized GLMM with a variable screening step. By including both the variable screening step and the lasso penalty, our two-step method successfully found associations that would have been missed when the original LassoGLMM retained no non-zero coefficients and when the non-penalized GLMM with all correlated variables failed due to the high number of variables correlated with each other.

### Comparison to categorical methods

To compare the performance of our method, which retains the continuous nature of the original measurements, to categorical methods, which remove the relative scale of the measurements and are used most frequently in microbiome studies, we dichotomized the original continuous measurements based on their mean values. For these newly dichotomized variables, we compared the genera abundance of the microbiota between the two groups using a Wilcoxon test [24].

**Fig. 5:**
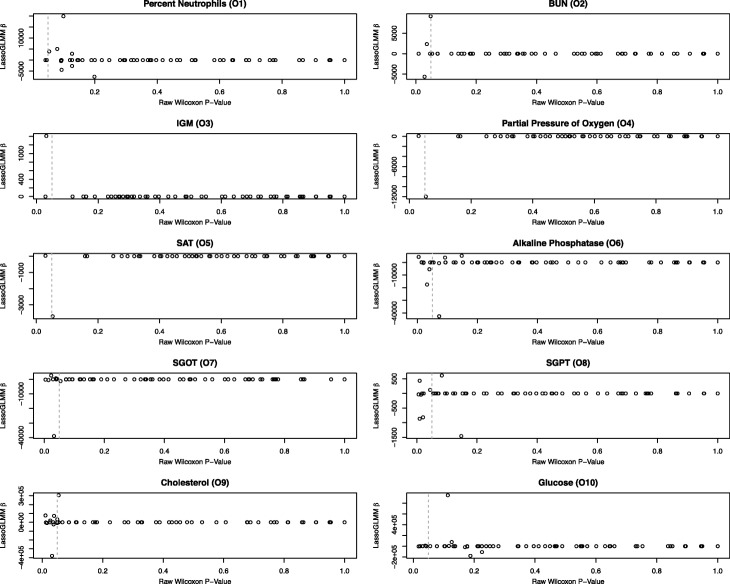
Wilcoxon *p*-values compared to LassoGLMM β coefficients for OC-COPD study. Each plot represents one LassoGLMM with non-zero coefficients. For each bacterial genus, the Wilcoxon p-value (before adjustment for multiple hypotheses testing) is plotted on the *X*-axis and the LassoGLMM β coefficient is plotted on the *Y*-axis. Most β coefficients are equal to zero. The dashed vertical line indicates nominal significance based on a Wilcoxon *P*-value of 0.05

**Fig. 6:**
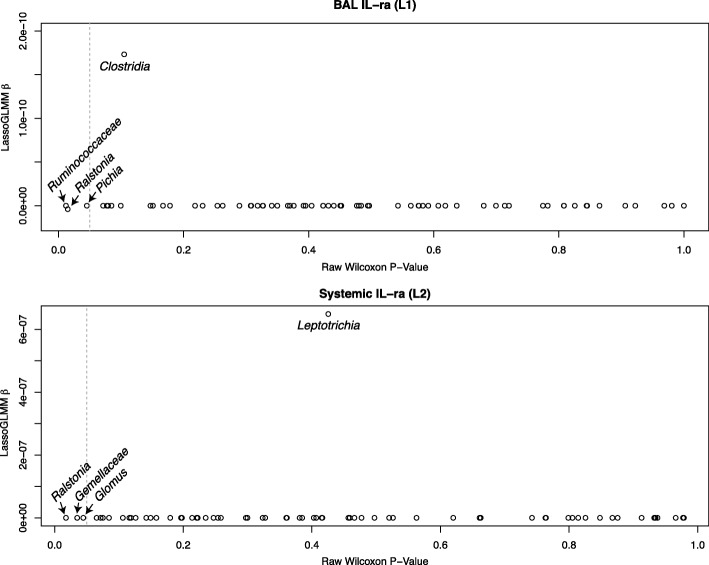
Wilcoxon p-values compared to LassoGLMM β coefficients for LHMP study. Each plot represents one LassoGLMM with non-zero coefficients. For each bacterial or fungal genera, the Wilcoxon p-value (before adjustment for multiple hypotheses testing) is plotted on the *X*-axis and the LassoGLMM β coefficient is plotted on the *Y*-axis. Most β coefficients are equal to 0; those that are not are labeled with their lowest taxonomic assignment appearing horizontally. The dashed vertical line indicates nominal significance based on a Wilcoxon p-value of 0.05. The nominally significant genera that have a β coefficient of 0 are labeled with their lowest taxonomic assignment appearing diagonally

For OC-COPD models O1 to O10, the genera that retained non-zero coefficients (suggesting a relationship between the genera and outcome) differed from the categorical method results. Bacterial genera (*n* = 1 to 12, mean = 5.4) were differentially abundant between above- and below-average clinical measurement outcome groups, before correcting for the large number of tests (Fig. 5). For the two cytokines that retained non-zero coefficients in our LassoGLMMs (models L1 and L2), dichotomized at the mean cytokine level, abundance levels for two bacterial genera and one fungal genus were significantly different (Fig. 6). After adjusting for multiple hypotheses testing using the Benjamini-Hochberg false discovery rate [25], no genera were significantly differentially abundant between above and below mean outcome or cytokine levels. Before multiple hypotheses testing correction, the 60 significantly different genera identified by the Wilcoxon tests across all 12 of the outcomes of interest showed 52% overlap with the 67 genera identified as strongly associated with the outcome by our two-step LassoGLMM method. With one exception (*Leptotricia* in model L2), all genera identified by our two-step LassoGLMM had an unadjusted Wilcoxon test *p*-value no greater than 0.23, suggesting a difference between the samples with high and low outcomes that is detectable by LassoGLMM but not by the simple Wilcoxon approach. The LassoGLMM is therefore more sensitive to associations, even in small sample sizes.

To show the impact of the cutoff value on determining associations, we examined using the first and third quartiles as thresholds for high/low abundances in the LHMP cytokine values. Using the first quartile cutoff, the Wilcoxon tests identified five genera as significantly differentially abundant (before multiple hypothesis correction) between high and low BAL IL-ra (Additional file 4: Figure S2a). These five genera have no overlap with the genera identified when the mean value was used as the cutoff, nor those identified by our LassoGLMM L1 model. Using the third quartile cutoff, the Wilcoxon tests identified two genera (Additional file 4: Figure S2b), including *Ralstonia*, which was identified using the mean value cutoff and by our LassoGLMM L1 model. For systemic IL-ra, the Wilcoxon tests identified the genus *Ralstonia* as significantly differentially abundant, regardless of cutoff value, but identified nine additional genera using the first quartile cutoff (Additional file 4: Figure S2c) and three additional genera using the third quartile cutoff (Additional file 4: Figure S2d). Both quartile-based cutoffs identified the genus *Prevotella* as differentially abundant, but this genus was not identified using the mean as the cutoff. No cutoff for high/low systemic IL-ra identified the association with *Leptotrichia* that was detected by our LassoGLMM L2 model.

## Discussion

We analytically identified associations between bacteria and fungi and continuous clinical variables, including standard blood chemistries as well as lung and peripheral cytokines. Our application of the two-step LassoGLMM approach to two clinical datasets represents an important addition to the field as it identifies relationships between microbes and repeatedly measured continuous outcomes while maintaining the outcomes as the response variables. We applied our two-step LassoGLMM approach to two mucosal microbiome datasets to analyze the relationship of microbes and their abundances to continuous clinically-related variables with repeated measurements.

Our aim was to explain variation and discover association between clinical variables and microbial abundance, with the exact value of this association being less important than the direction of the association. Explanatory associations, such as those predicted by our LassoGLMM method, are more useful in hypothesis generation than in predicting future events or values given our small sample sizes (7 of our 12 models were considered overfitted, which would be a problem if predictive modeling were our aim [26]). For both the oral and lung microbiomes, testing the hypotheses based on our identified associations remains elusive at this time. In smaller-scale or more well-known systems, associations identified with the LassoGLMM method could likely be validated in the laboratory.

Traditionally, associations between microbial abundance and continuous clinically-related outcomes, with repeated measures or not, have been built on grouping samples using arbitrary cutoffs of clinical values measured within the study itself. In our comparison of our two-step LassoGLMM to the Wilcoxon test, we mimicked a common cutoff for comparison (above/below study mean) to define a study sample group and briefly explored how changing that cutoff can lead to different results. Because the analytic comparison groups are often defined by the dichotomization of a variable, the cutoff point is study sample dependent, and the choice of this cutoff point impacts the results, there are limitations on reproducibility outside the current study and the range of a measure’s natural variation in the larger population is ignored. Any association between microbial abundances and a repeated clinical measurement found by this type of test (Wilcoxon) ignores the fact that repeated samples are not independent of each other. This limitation may explain why there was minimal overlap between the genera identified by our two-step LassoGLMMs and the Wilcoxon test.

Repeated measurements taken in the clinic introduce a data structure that violates a number of assumptions among common statistical tests, even those developed specifically for microbiome studies. Multivariate Association with Linear Models (MaAsLin) was recently developed to simultaneously find associations between microbes and multiple clinical outcomes, including continuous variables, through variable selection and linear modeling [27]. However, MaAsLin models do not allow for repeated measurements and their complex covariance structure because MaAsLin requires that all samples be independent, originating from different subjects. Another approach is the two-part zero-inflated Beta regression model with random effects (ZIBR), which can handle repeated measurements through the use of random effects. However, ZIBR assumes that all subjects will have samples taken at the same time points with no missing measurements [28]. Real-world datasets, including ours, rarely contain all time points for all subjects and may have missing data for various reasons including missed appointments or failed amplification and sequencing. A third approach, negative binomial mixed models (NBMM) is more similar to our method in that it uses mixed models to handle complex correlation structures and can handle missing data [29]. However, none of these other methods allow for correlations or interactions between microbial abundances since microbial measures are restricted to being the response variable only.

Our use of the LassoGLMM takes advantage of its ability to account for correlations between genera, which may be indicative of biological interactions. Too many interactions or correlations between genera can be problematic for the lasso penalty, as it may discard a biologically important genus while retaining a non-zero coefficient for a correlated but less biologically important genus. We mitigate this problem by reducing the number of genera entered into the LassoGLMM with a variable screening step. The “choices” that the lasso penalty makes highlight the need for future study of the relationships between the genera in addition to their relationships with the outcome variable. Genera whose coefficients are pushed to zero may be chemically or physically interacting with genera whose coefficients are non-zero. Or, if negatively correlated with each other, may be performing the same function. This biological redundancy may stem from bacterial interactions or from competition to fill the same niche. Biological interactions between genera within a microbiome represent an area of active research and in the meantime, methods such as our two-step LassoGLMM that can account for these uncharacterized interactions should be better able to determine associations than methods that ignore them.

## Conclusions

The potential applications of our two-step LassoGLMM are multiple and go beyond what we have used it for in our study. We took advantage of the ability to account for potentially confounding categorical variables, treatment assignment and gender in OC-COPD, and HIV status and smoking status in LHMP. This ability can be used to account for attributes that are known or suspected to influence the outcome variable, including host genotype. We made use of the ability to analyze repeated measurements from the same individual, over two time points in OC-COPD, and in two lung locations (right and left lungs were sampled separately) in the LHMP. The method can accommodate any number of repeated measurements, including long-term longitudinal studies, even when the number of measurements per individual is not identical. The inclusion of the individual as a random effect also accounts for an uneven number of observations per subject, a common issue in the clinic where study participants can be followed for different lengths of time, can be “lost to follow-up”, may die, or may drop out of the study. The generalized nature of our two-step LassoGLMM also allows for the analysis of variables that do not follow a normal distribution, including time-to-event and categorical outcomes. The lasso penalty allows for variable selection to select the strongest genera associations but the selection criteria may be influenced by the correlations between microbes inherent in relative abundance and other compositional data. However, our two-step LassoGLMM is not limited to relative abundance data and when a consensus is reached about the optimal normalization or transformation methods for microbiome data, this method will be able to handle that data and improve its performance.

We have demonstrated that our two-step version of the lasso-penalized generalized linear mixed model can be applied to microbiome studies with continuous outcomes and repeated measures. This model works well with both 16S rRNA gene surveys and more complicated 16S/ITS combination studies. The method combines the well-established lasso penalty to account for the large number of variables with the mixed model to account for repeated sampling—including longitudinal studies—and other variables that are known to be associated with the outcome. The addition of a variable screening step ensures that models for more outcome variables are solvable than with a single step LassoGLMM. The power of our two-step LassoGLMM lies not only in its ability to identify known associations between microbes and continuous clinical variables, but in its ability to identify novel associations that can be used to test new potential biomarkers.

## Additional files

Additional file 1: **Table S1.** Correlations between bacteria and laboratory measurements in OC-COPD. Table displays the Spearman correlations between all bacteria-laboratory measurement pairs. *P* values were adjusted (AdjustedP) using the Bonferroni correction. The last column (NwithGenus) is a count of the number of samples that contained the genus in that correlation-pair. (XLSX 173 kb)

Additional file 2: **Figure S1.** Additional OC-COPD associations between laboratory measurements and bacteria identified by LassoGLMM. Strong associations between bacteria and (**a**) percent neutrophils (O1), (**b**) partial pressure of oxygen PO2(O4) (**c**) SAT (O5), (**d**) alkaline phosphatase (O6), and (**e**) glucose (O10). Each horizontal grey line represents an individual. When a colored circle is located on the grey line, it is the relative abundance of that microbe for that subject. Perfect positive association between clinical variable and bacteria would form a line from the bottom-left to the top-right of the graph and would have a highly positive β coefficient in the LassoGLMM. Perfect negative association would form a line from the top-left to the bottom-right of the graph and would have a highly negative β coefficient. (PDF 287 kb)

Additional file 3: **Table S2.** Correlations between bacteria or fungi and cytokines in LHMP. Table displays the Spearman correlations between all bacteria/fungi-cytokine pairs. *P* values were adjusted (AdjustedP) using the Bonferroni correction. The last column (NwithGenus) is a count of the number of samples that contained the genus in that correlation-pair. (XLSX 161 kb)

Additional file 4: **Figure S2.** Wilcoxon *p*-values based on quartile cutoffs compared to LassoGLMM β coefficients for LHMP study. Each plot represents one LassoGLMM with non-zero coefficients. For each bacterial or fungal genera, the Wilcoxon p-value (before adjustment for multiple hypotheses testing) based on the first (a and c) or third quartile (b and d) is plotted on the *X*-axis and the LassoGLMM β coefficient is plotted on the *Y*-axis. Most β coefficients are equal to 0; those that are not are labeled with their lowest taxonomic assignment appearing horizontally. The dashed vertical line indicates nominal significance based on a Wilcoxon p-value of 0.05. The nominally significant genera that have a β coefficient of 0 are labeled with an arrow indicating their lowest taxonomic assignment, except in panel c where they would be, from smallest to largest p-value, *Catonella, Actinomyces, Porphyromonas, Alicyclobacillus, Megasphaera, Ramularia, Prevotella, Ralstonia, Atopobium,* and *Veillonella*. (PDF 246 kb)

## Abbreviations

16S rRNA gene: Gene that codes for the 16S component of the small subunit of the ribosome
BAL: Bronchoalveolar lavage
BIC: Bayesian information criterion
COPD: Chronic obstructive pulmonary disease
GLM: Generalized linear model
GLMM: Generalized linear mixed model
IL-RA: Interleukin receptor antagonist
ITS: Internal transcribed spacer
LassoGLMM: Lasso-penalized generalized linear mixed model
LHMP: Lung HIV microbiome project
LMM: Linear mixed model
MaAsLin: Multivariate Association with Linear Models
NBMM: Negative binomial mixed model
OC-COPD: Oral Cyclosporine in Chronic Obstructive Pulmonary Disease
QIIME: Quantitative insights into microbial ecology
SD: Standard deviation
ZIBR: Zero-inflated beta regression
