# Supplementary figures and images for: Measuring associations between the microbiota and repeated measures of continuous clinical variables using a lasso-penalized generalized linear mixed model
Source: BioData Min. 2018 Jun 15;11:12. doi: 10.1186/s13040-018-0173-9 (PMC6003033; doi:10.1186/s13040-018-0173-9)

(a)

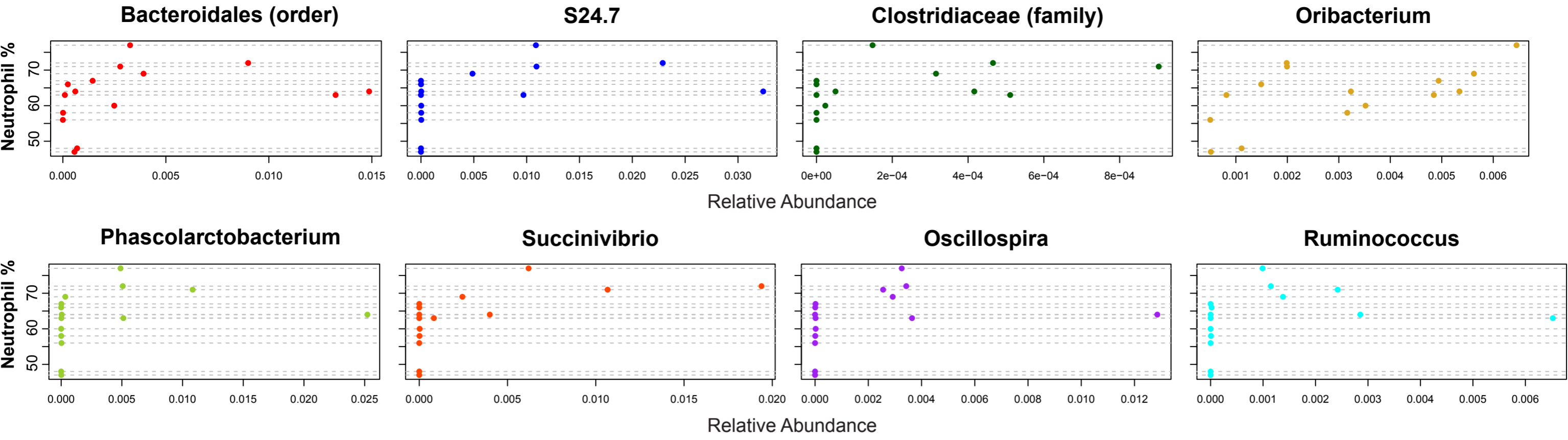

(b)

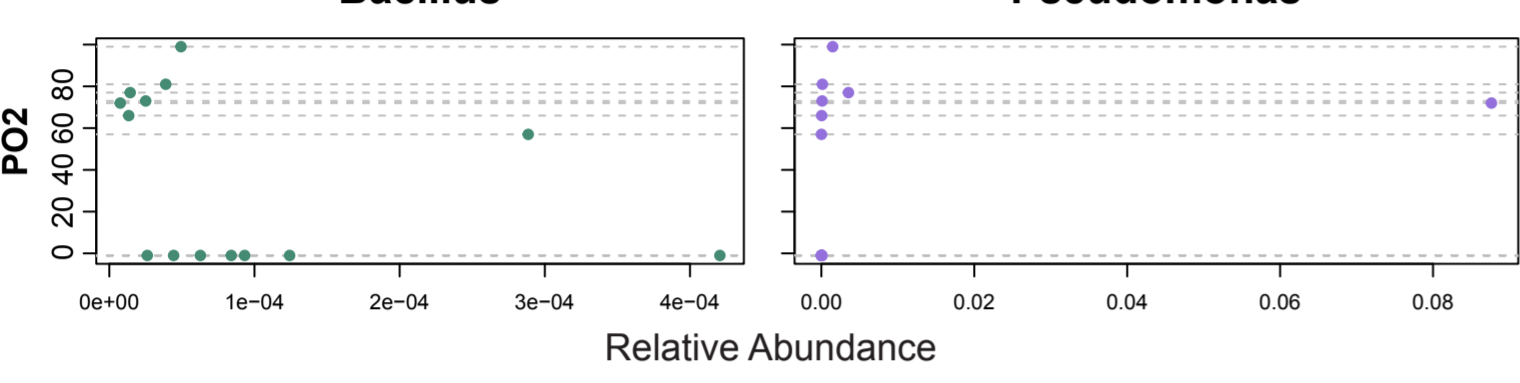

(c)

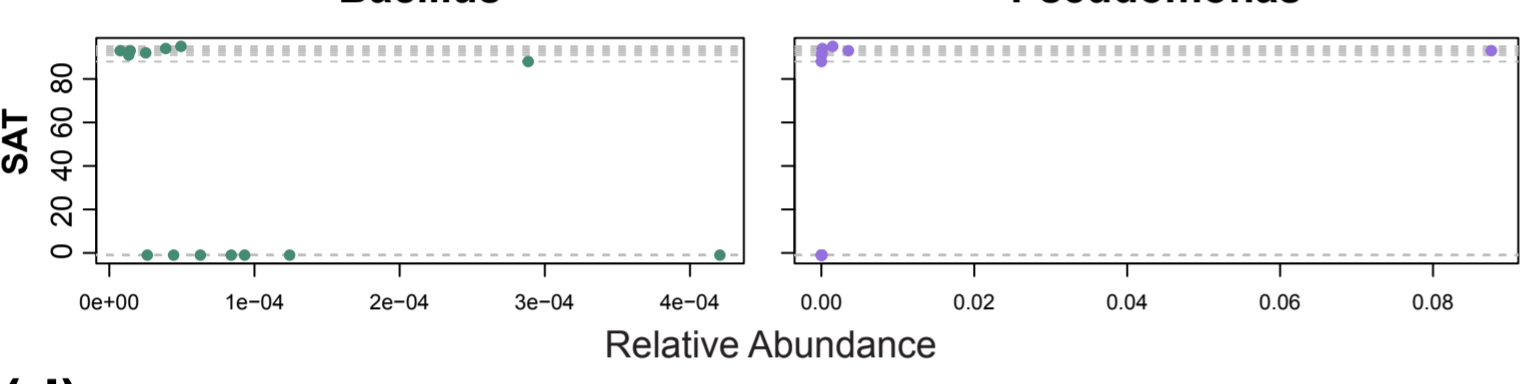

(d)

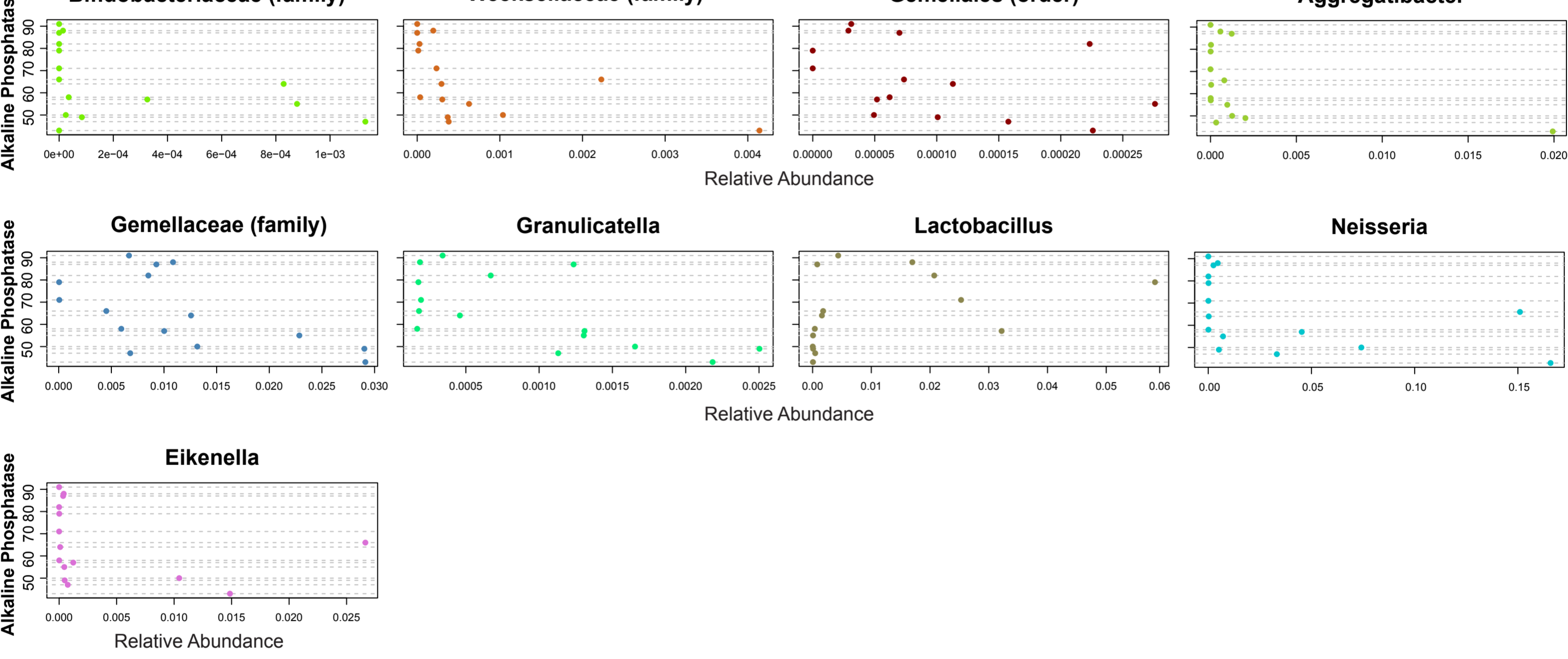

(e)

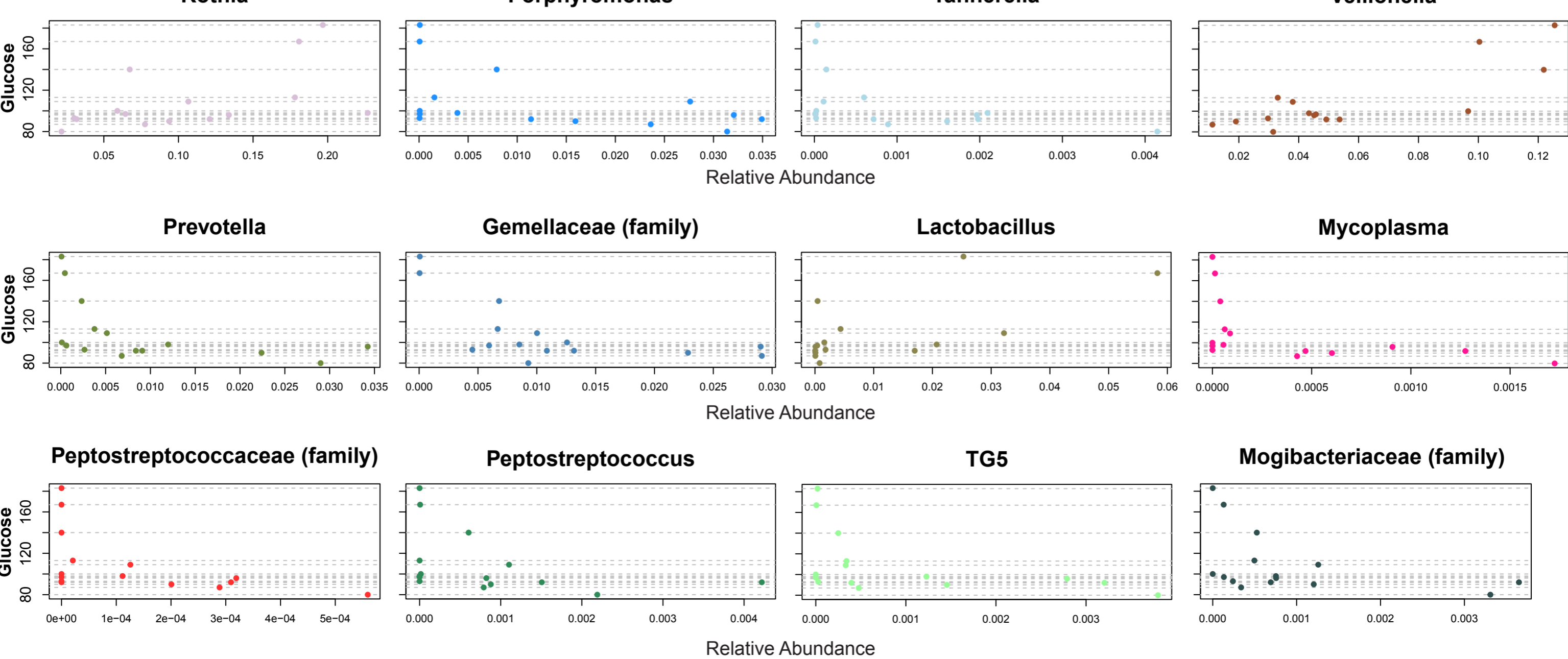

Supplement: Supplementary file 2 — Figure S1. Additional OC-COPD associations between laboratory measurements and bacteria identified by LassoGLMM. Strong associations between bacteria and (a) percent neutrophils (O1), (b) partial pressure of oxygen PO2(O4) (c) SAT (O5), (d) alkaline phosphatase (O6), and (e) glucose (O10). Each horizontal grey line represents an individual. When a colored circle is located on the grey line, it is the relative abundance of that microbe for that subject. Perfect positive association between clinical variable and bacteria would form a line from the bottom-left to the top-right of the graph and would have a highly positive β coefficient in the LassoGLMM. Perfect negative association would form a line from the top-left to the bottom-right of the graph and would have a highly negative β coefficient. (PDF 287 kb) [file 13040_2018_173_MOESM2_ESM.pdf]

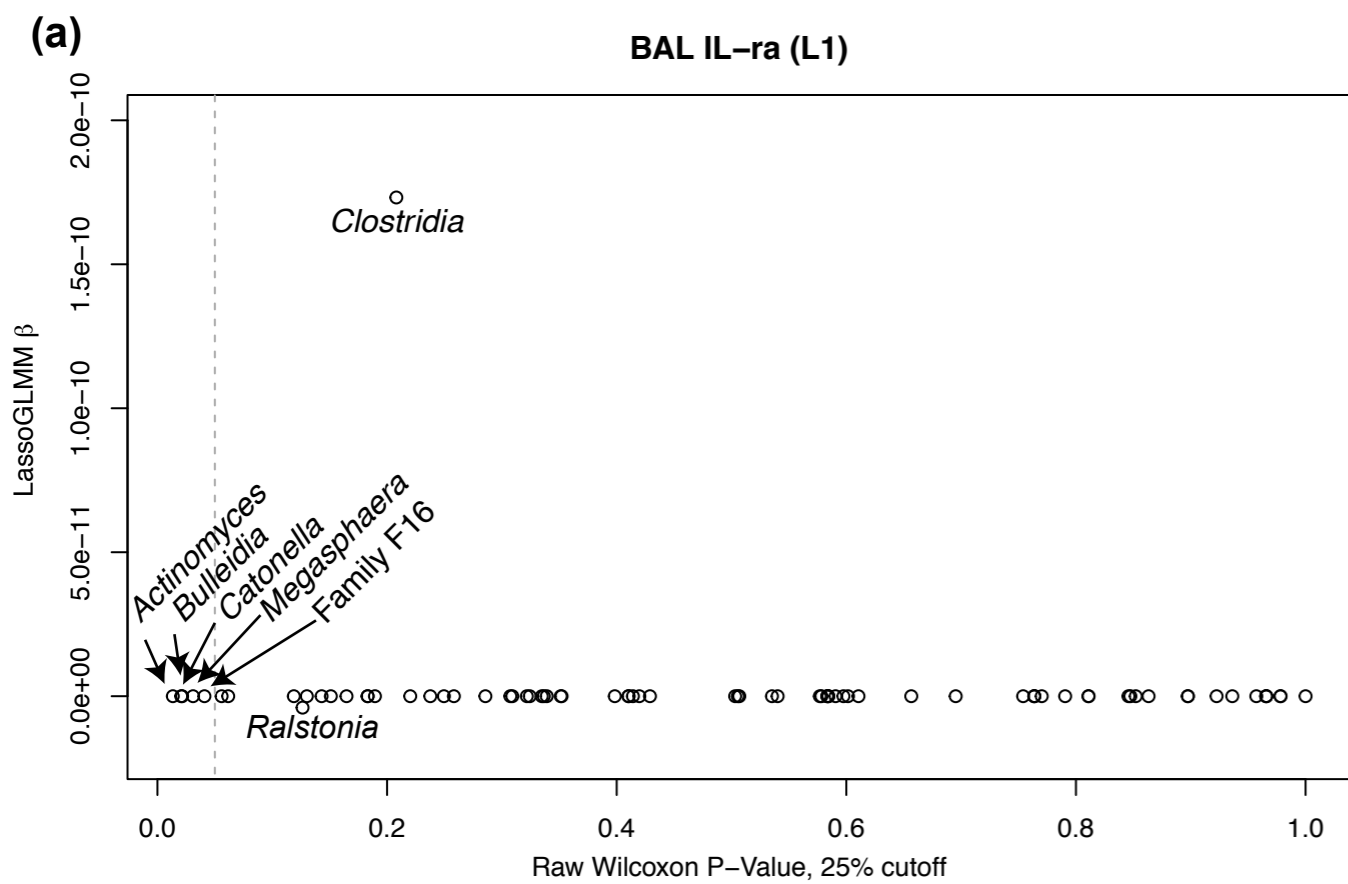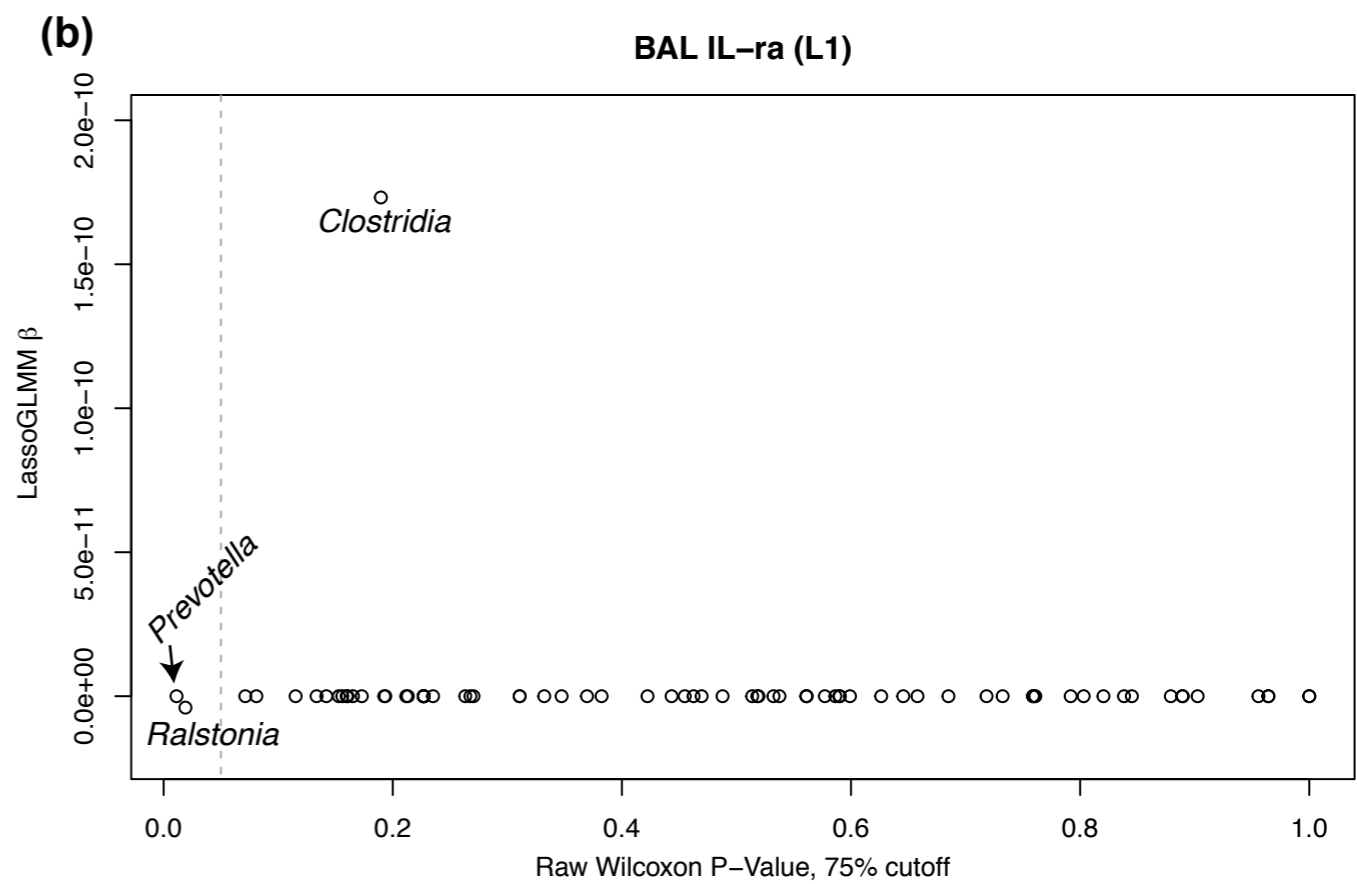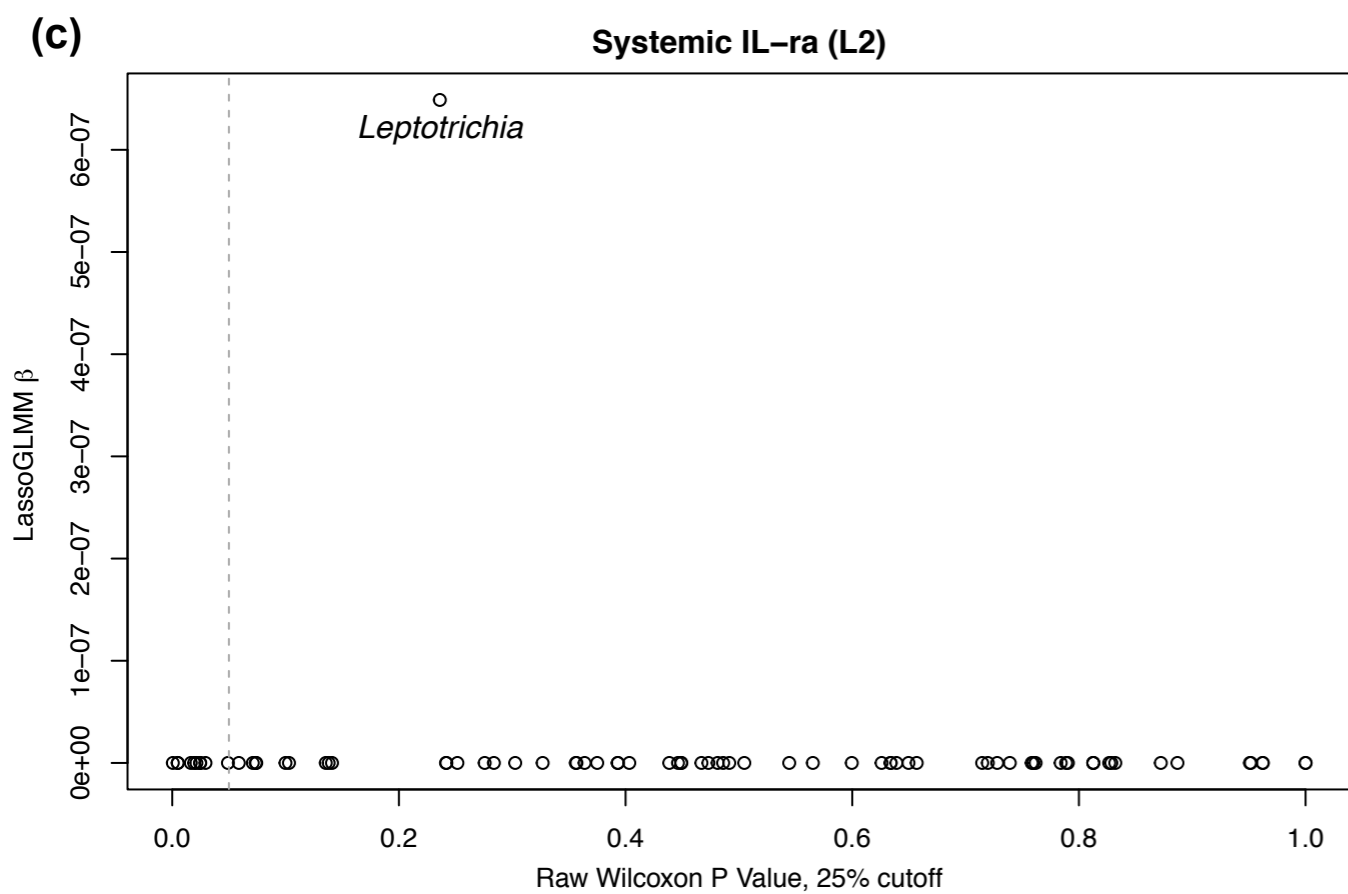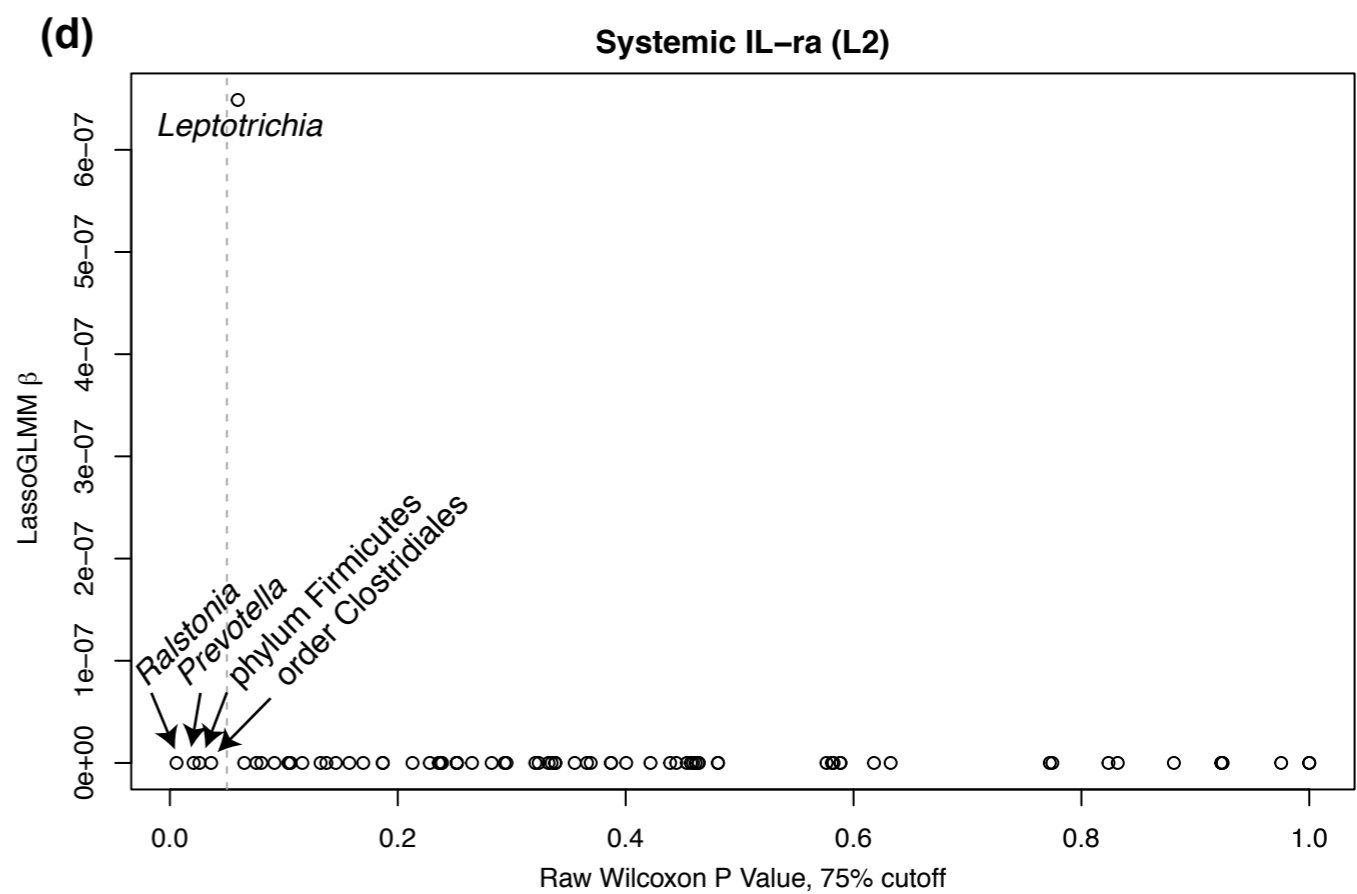

Supplement: Supplementary file 4 — Figure S2. Wilcoxon p-values based on quartile cutoffs compared to LassoGLMM β coefficients for LHMP study. Each plot represents one LassoGLMM with non-zero coefficients. For each bacterial or fungal genera, the Wilcoxon p-value (before adjustment for multiple hypotheses testing) based on the first (a and c) or third quartile (b and d) is plotted on the X-axis and the LassoGLMM β coefficient is plotted on the Y-axis. Most β coefficients are equal to 0; those that are not are labeled with their lowest taxonomic assignment appearing horizontally. The dashed vertical line indicates nominal significance based on a Wilcoxon p-value of 0.05. The nominally significant genera that have a β coefficient of 0 are labeled with an arrow indicating their lowest taxonomic assignment, except in panel c where they would be, from smallest to largest p-value, Catonella, Actinomyces, Porphyromonas, Alicyclobacillus, Megasphaera, Ramularia, Prevotella, Ralstonia, Atopobium, and Veillonella. (PDF 246 kb) [file 13040_2018_173_MOESM4_ESM.pdf]
